# Thromboprophylaxis Reduced Venous Thromboembolism in Sickle Cell Patients with Central Venous Access Devices: A Retrospective Cohort Study

**DOI:** 10.3390/jcm11051193

**Published:** 2022-02-23

**Authors:** Stéphanie Forté, Gonzalo De Luna, Jameel Abdulrehman, Nafanta Fadiga, Olivia Pestrin, Anne-Laure Pham Hung d’Alexandry d’Orengiani, John Chinawaeze Aneke, Henri Guillet, Dalton Budhram, Anoosha Habibi, Richard Ward, Pablo Bartolucci, Kevin H. M. Kuo

**Affiliations:** 1 Division of Medical Oncology and Hematology, University Health Network, Toronto, ON M5G 2C4, Canada; stephanie.forte@umontreal.ca (S.F.); jameel.abdulrehman@uhn.ca (J.A.); nafanta.fadiga@queensu.ca (N.F.); olivia.pestrin@gmail.com (O.P.); anekejc@gmail.com (J.C.A.); dbudhram@qmed.ca (D.B.); richard.ward@uhn.ca (R.W.); 2 Sickle Cell Referral Center, Department of Internal Medicine, Henri Mondor University Hospital, UPEC, APHP, 94000 Créteil, France; gonzalodeluna@gmail.com (G.D.L.); anne-laure.phamhung-dalexandrydorengi@aphp.fr (A.-L.P.H.d.d.); henri.guillet@aphp.fr (H.G.); anoosha.habibi@aphp.fr (A.H.); pablo.bartolucci@aphp.fr (P.B.); 3 Laboratoire D’Excellence, GRex, Institut Mondor, INSERM U955 Equipe 2, 94000 Créteil, France

**Keywords:** anemia, sickle cell, central venous catheters, upper extremity deep vein thrombosis, vascular access devices, venous thromboembolism

## Abstract

Sickle cell disease (SCD) induces a chronic prothrombotic state. Central venous access devices (CVADs) are commonly used for chronic transfusions and iron chelation in this population. CVADs are an additional venous thromboembolism (VTE) risk factor. The role of thromboprophylaxis in this setting is uncertain. The objectives are: (1) to determine whether thromboprophylaxis reduces VTE risk in SCD patients with CVAD and (2) to explore characteristics associated with VTE risk. We identified adults with SCD and CVAD intended for chronic use (≥3 months) at two comprehensive SCD centers. Thromboprophylaxis presence; type; intensity; and patient-, catheter-, and treatment-related VTE risk factors were recorded. Among 949 patients, 49 had a CVAD (25 without and 24 with VTE prophylaxis). Thromboprophylaxis type and intensity varied widely. Patients without thromboprophylaxis had higher VTE rates (rate ratio (RR) = 4.0 (95% confidence interval: 1.2–12.6), *p* = 0.02). Hydroxyurea was associated with lower VTE rates (RR = 20.5 (6.4–65.3), *p* < 0.001). PICC lines and Vortex and Xcela Power implantable devices were associated with higher rates compared with Port-a-Cath (RR = 5.8 (1.3–25.9), *p* = 0.02, and RR = 58.2 (15.0–225.0), *p* < 0.001, respectively). Thromboprophylaxis, hydroxyurea, and CVAD subtype were independently associated with VTE. The potentially protective role of thromboprophylaxis and hydroxyurea for VTE prevention in patients with SCD and CVAD merits further exploration.

## 1. Introduction

Sickle cell disease (SCD) is a high-risk inherited thrombophilia [1]. The abnormal sickle hemoglobin polymerizes under hypoxic conditions, leading to vaso-occlusion and intravascular hemolysis, which in turn activate the coagulation cascade and platelets, while impairing fibrinolysis, culminating in a chronic hypercoagulable state [1,2,3,4]. The cumulative incidence of venous thromboembolism (VTE) is reported to be 11% by age 40 with high rates of recurrence (>24% at 5 years) [5,6,7].

SCD patients often require central venous access devices (CVADs) for chronic transfusions and/or iron chelation. Unfortunately, CVADs are known to be highly prothrombotic with a catheter-related thrombotic rate of 24% in SCD patients [8].

While it is well established that those with SCD and CVAD are at high risk for VTE, the role of pharmacological thromboprophylaxis for the prevention of VTE in this setting is uncertain due to a paucity of data. As a result, the practice is heterogeneous both across and within centers. We hypothesized that thromboprophylaxis would reduce the VTE rate in adult SCD patients with CVAD. Our primary objective was to test the association between the presence of thromboprophylaxis and VTE occurrence in this population. Our secondary objective was to explore additional potential VTE risk factors in this setting.

## 2. Materials and Methods

The study was reviewed and approved by the ethics committee of the two participating centers.

### 2.1. Study Design

This multicenter retrospective cohort study assessed SCD patients with CVAD from two SCD reference centers: the Red Blood Cell Disorders (RBCD) Clinic, University Health Network (UHN), Toronto, Canada, from 1 January 2009 to 31 December 2017, and Unité des Maladies Génétiques du Globule Rouge (UMGGR), Hôpitaux Universitaires Henri Mondor, Créteil, France, from 1 January 2016 to 5 February 2020. To identify patients who had a CVAD during the observation period, at UHN, 4 patient databases (diagnostic imaging, thrombosis clinic, RBCD clinic, and apheresis unit) were cross-referenced, and at UMGGR, the list of all patients in the medical day unit was screened.

### 2.2. Inclusion and Exclusion Criteria

Adult SCD patients (≥18 years old, any sickling genotype (SS, S^0^, SC, S^+^, and any other variant sickling genotype)) with a CVAD for chronic use (≥3 months) were included. Patients were excluded if the CVAD was inserted before the observation period or if information about the date, type, and/or duration of insertion was missing.

### 2.3. Patient Characteristics

Patient-, catheter-, device-, and treatment-related risk factors of VTE at the time of line insertion were extracted from electronic patient records. Patient-related risk factors included age, sex, sickle genotype, body mass index (BMI), previous history of VTE, and known inherited or family history of thrombophilia. Transient VTE risk factors included medium- or high-risk surgery (as defined in the TAPS protocol [9]), pregnancy, estrogen-based hormonal contraception, active cancer or chemotherapy, sepsis, or line infection at any time while the CVAD was in place. Device-related factors included the site of insertion (jugular, brachial), type of CVAD (peripherally inserted central catheter (PICC) line, tunneled catheters (e.g., Hickman), subcutaneous port, subtype of CVAD (e.g., Port-a-Cath, Vortex)), and duration of CVAD. Treatment-related risk factors included indication for CVAD insertion, type of transfusion (simple, partial manual exchange, or automated exchange), and ferritin and hydroxyurea (HU) use.

### 2.4. Exposure

The presence, type, and intensity of thromboprophylaxis at the time of CVAD insertion was collected. The same parameters were collected again at the time of VTE. If the patient did not have a VTE, these parameters were recorded at the time of CVAD removal or the end of the study period, whichever was earlier.

Thromboprophylaxis at treatment dose was defined as either low molecular weight heparin (LMWH), direct oral anticoagulant (DOAC) at approved treatment dosing, or warfarin with a target international normalized ratio (INR) of 2.0–3.0. Thromboprophylaxis at reduced dosing was defined as either LMWH, direct oral anticoagulant (DOAC) at approved prophylactic dosing, or warfarin with a target of INR 1.5–2.5, or <2.0, or acetylsalicylic acid (ASA) at any dose.

### 2.5. Outcome

VTE was defined as catheter-related thrombosis (CRT), right atrial or ventricular thrombus, proximal and distal lower extremity deep vein thrombosis (DVT), segmental pulmonary embolism (PE), or VTE at other sites, confirmed by diagnostic imaging report. CRT was further defined as a thromboembolism in the same upper (or proximal to axillary vein) or lower limb (or proximal to popliteal vein) as the CVAD. Both symptomatic and incidental VTE were included in the outcome definition. A VTE event was captured if it occurred while the CVAD was in place during the study period. Only the incident event was counted. Individual patients’ observation time (or time to exit) was defined as the time from CVAD insertion to the time of VTE, CVAD removal, or end of observation period, whichever occurred first, regardless of CVAD change within the interval. A record of major bleeding (as defined by ISTH guidelines) was made [10].

### 2.6. Statistical Analyses

Descriptive statistics were used to present the patient characteristics at the time of CVAD insertion. VTE incidence rates were calculated. The 95% confidence intervals (CI) were generated using the Poisson distribution. As this is a hypothesis-generating study, we included a host of potential risk factors in the analysis. A Poisson regression was used to compare incidence rates of VTE according to a priori defined potential risk factors (thromboprophylaxis, age, sex, genotype, CVAD type, location, subtype, BMI, ferritin, additional VTE risk factors, and hydroxyurea). Continuous variables were recoded as categorical variables for the purposes of the time-to-VTE analysis. Time-to-VTE was used as the offset variable. Statistical significance was defined as *p* < 0.05. The presence of thromboprophylaxis, additional VTE risk factors, sex, and any variable with *p* < 0.2 on univariate analysis were used to build a multivariable Poisson regression model of VTE incidence. IBM SPSS Version 26 was used (IBM Corp. Released 2019. IBM SPSS Statistics for Windows, Version 26.0. IBM Corp: Armonk, NY, USA).

## 3. Results

**Figure 1:**
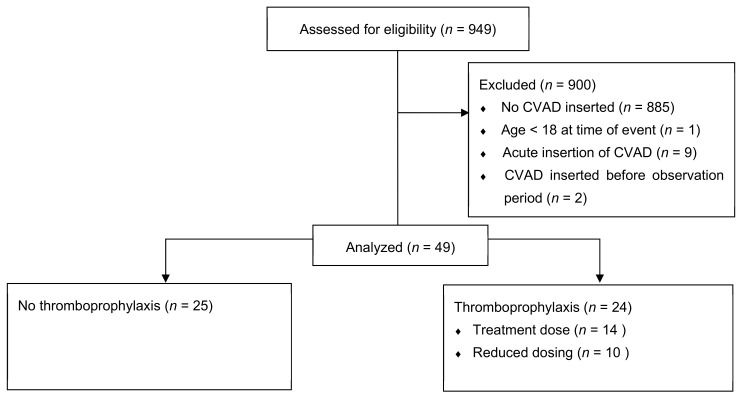
CONSORT 2010 Flow Diagram.

At UHN and CHU Henri-Mondor, 949 SCD patients were screened for eligibility, and 49 met the inclusion criteria (Figure 1). At the time of CVAD insertion, 25 received no VTE thromboprophylaxis, and 24 received VTE thromboprophylaxis. In the latter group, 10 (42%) received reduced dosing, and 14 (58%) received treatment dose anticoagulation. 

Mean age was 33.8 (standard deviation (SD), 9.1) and 31.9 (9.1) years in those without and with thromboprophylaxis, respectively (Table 1). Majority were female (16 (64%) and 17 (71%), respectively). Hydroxyurea therapy was used by most patients (17 (68%) and 12 (50%), respectively). Subcutaneous port was the preferred CVAD in both groups (23 (92%) and 20 (83%), respectively), while only a few patients had PICC lines (2 (8%) and 4 (17%), respectively). The median duration of CVAD insertion was 2.3 (range: 0.2–8.9) and 3.5 (0.3–8.5) years, respectively. The type of thromboprophylaxis varied widely but remained stable for the duration of CVAD implantation, except for 2 patients on warfarin, where the target INR increased from 1.5–2.5 to 2.0–3.0.

The observation periods were 62.2 and 86.2 patient-years in the no thromboprophylaxis and thromboprophylaxis groups, respectively (Table 2). VTE was captured in 10 (40%) and 4 (17%) patients, respectively. The incidence rates of VTE were 0.44 (95% CI: 0.28–0.70) and 0.13 (0.05–0.32) events per 1000 CVAD implantation days, respectively. The types of VTE in patients without thromboprophylaxis varied with regard to location and severity: 3 upper extremity CRT, 1 PE, 4 right atrial thrombi, and 3 thrombi from other sites (jugular, subclavian, inferior vena cava). The patient with a subclavian vein thrombosis had a concomitant PE. One patient with a right atrial thrombus underwent open heart surgery to remove the clot due to an initial misdiagnosis as a myxoma. Another patient with right atrial thrombus initially refused anticoagulation and subsequently developed a PE after 11 months. In the patient with the right internal jugular vein clot, a PE developed after 2 months of anticoagulation. In the thromboprophylaxis group, we observed 3 upper extremity CRT and 1 internal jugular vein thrombosis. There was no major bleeding or thrombosis- or hemorrhage-related deaths.

On univariate analysis, the presence of thromboprophylaxis was associated with a 4.0 (1.2–12.6) relative reduction in the rate of VTE (*p* = 0.02) (Table 3). Hydroxyurea was associated with a 20.5-fold reduction of the VTE rate ((6.4–65.3), *p* < 0.001). PICC line and Vortex or Xcela Power implantable CVADs were associated with higher rates of VTE compared with traditional Port-a-Cath (RR = 5.8 (1.3–25.9), *p* = 0.02, and RR = 58.2 (15.0–225.0), *p* < 0.001, respectively). However, age, sex, CVAD type and location, BMI, ferritin, and additional VTE risk factors (including prior VTE) were not significantly associated with the risk of VTE occurrence (all *p* > 0.05). Only 4 patients with a non-SS/Sβ^0^ genotype were present in the study, precluding analysis by sickling genotype.

On multivariable analysis, after adjustment for sex, age, additional VTE risk factors, hydroxyurea, thromboprophylaxis, BMI, and CVAD subtype, the relative rate reduction of VTE with thromboprophylaxis was 14.9 (2.0–108.7) (*p* = 0.01) (Table 3). In this model, hydroxyurea was also strongly associated with a reduced rate of VTE events (RR = 47.1 (8.0–276.0) (*p* < 0.001)). The presence of a PICC line or Vortex or Xcela Power was still independently associated with a higher rate of VTE compared with Port-a-Cath (RR = 34.7 (3.5–343.6), *p* = 0.02, and RR = 20.3 (3.8–107.9), *p* < 0.001, respectively).

## 4. Discussion

This is the first study exploring the role of VTE thromboprophylaxis in SCD patients with CVAD. We show a rate reduction of VTE events in patients on some form of thromboprophylaxis. However, we observed considerable heterogeneity in the type and intensity of thromboprophylaxis used by physicians and, therefore, cannot conclude on an optimal strategy. This variability in clinical practice reflects physician and patient preference in the context of lack of evidence and variable drug accessibility. A Canadian physician survey showed that 42% of SCD experts were not very confident or not at all confident in the choice of prophylaxis. While 50% did not routinely use thromboprophylaxis, 17% did use DOAC at a reduced dosing [11].

Overall, our VTE rates of 0.44 and 0.13 events/1000 CVAD implantation days for those without and with thromboprophylaxis were in the lower range compared with published studies in this population. Reported rates of CVAD-related thrombosis in patients with thromboprophylaxis ranged from 0.16 to 0.99 events/1000 implantation days [12,13,14,15,16,17,18]. This may be explained by the difference in types of CVAD used. Nontunneled CVADs were associated with the highest rates of VTE [14]. These only represented a minority of CVADs in our study, but were highly associated with a risk of VTE. Higher rates of infections and more frequent changes of devices may explain this increased risk [18]. To further explain the relatively moderate incidence of VTE in our cohort, we speculate that the frequent coadministration of hydroxyurea with transfusions could have been protective. In fact, hydroxyurea was independently associated with a 47.1 (8.0–276.0)-fold reduction of the VTE rate.

Published evidence on the effect of hydroxyurea on hemostasis is inconsistent. One retrospective study suggested that hydroxyurea was associated with a higher rate of VTE on multivariable analysis, probably reflecting additional patient risk factors, especially given the biological data showing benefit on thrombin markers [19]. On the other hand, patients who were taking hydroxyurea had lower plasma mRNA tissue factor (TF) and thrombin generation marker levels [20,21]. Additionally, hydroxyurea reduces sickling of red blood cells (RBCs), release of free hemoglobin, leukocytosis, and thrombocytosis, which in turn are known to contribute to a prothrombotic state [4,22]. The association of hydroxyurea with a reduced risk of VTE could be mediated by decreased hospitalizations.

The high rate of atrial thrombi (8%) in our cohort was concerning, but their discovery may be related to routine use of echocardiography every 1 to 2 years for screening of pulmonary hypertension for all sickle cell patients in both centers. The type of CVAD used may also explain this high prevalence. All atrial thrombi were diagnosed in patients with Vortex^®^ and Xcela Power^®^ CVAD. Similarly, Brewin and colleagues observed asymptomatic atrial thrombi in 10% of patients with Vortex LP^®^ [8]. Atrial thrombi are an under-recognized and potentially fatal consequences of CVAD, with an estimated prevalence of up to 18% in patients on hemodialysis [23,24,25]. The positioning of the tip of the CVAD in the right atrium could favor the development of such thrombi [25].

To our knowledge, this is the first study to provide evidence on the use of thromboprophylaxis in SCD patients with CVAD. It is also the largest cohort of VTE in adult SCD patients with CVAD for chronic use. Limitations to our study include its retrospective nature and heterogeneity in the choice of anticoagulation. Our study could not formally assess nonmajor bleeding outcomes, as these were not systematically collected during routine visits. However, based on a large population-based study by Hariharan and colleagues, we would expect some increased risk of bleeding, especially when on treatment dose anticoagulation [26]. Additionally, some information about VTE risk factors throughout the observation period could have been missed if care episodes occurred in outside centers. Finally, additional confounders, such as acute illness, could have biased the results.

## 5. Conclusions

In conclusion, the results are highly suggestive of the protective effect of thromboprophylaxis and hydroxyurea against thrombosis in patients with SCD and CVAD. However, the magnitude of the effect size requires confirmation, and the best modalities of pharmacological thromboprophylaxis require further studies. A pilot randomized controlled trial is underway to prospectively validate these findings.

## Figures and Tables

**Table 1:** Baseline demographics of adult sickle cell patients with central venous access device intended for long-term use. Characteristics of patients at the time of line insertion are shown. VTE risk factors were captured throughout the time of CVAD insertion.

			Thromboprophylaxis	
Variable			No (*n* = 25)	Yes (*n* = 24)
Age, years (mean (SD))			33.8 (9.1)	31.9 (9.1)
	<30 years		10 (40)	13 (54)
	30 to <40 years		8 (32)	7 (29)
	≥40 years		7 (28)	4 (17)
Sex, *n* (%)	Male		9 (36)	7 (29)
	Female		16 (64)	17 (71)
BMI, kg/m^2^ (mean (SD))			22.4 (4.2)	24.4 (3.9)
	BMI < 25 kg/m^2^, *n* (%)		21 (84)	14 (64)
	BMI ≥ 25 kg/m^2^, *n* (%)		4 (16)	8 (36)
Genotype, *n* (%)	SS		24 (96)	20 (83)
	SC		1 (4)	3 (13)
	Sβ^0^		0	1(4)
Main indication for CVAD insertion, *n* (%)	Transfusion			
		Secondary stroke prevention	0	7 (29)
		Primary stroke prevention	2 (8)	1 (4)
		Vasculopathy, moyamoya-like	5 (20)	3 (13)
		Priapism	1 (4)	1 (4)
		Pain	5 (20)	4 (17)
		Secondary prevention of acute chest syndrome	2 (8)	0
		Solid organ allograft protection	1 (4)	1 (4)
		Pregnancy	2 (8)	1 (4)
		Leg ulcer	1 (4)	1 (4)
		Pulmonary hypertension	2 (8)	4 (17)
		Other	3 (12)	0
	Venous access		0	1 (4)
	Intravenous iron chelation		1 (4)	0
Type of transfusion, *n* (%)	Partial manual		3 (12)	9 (38)
	Automated		19 (76)	14 (58)
	Simple transfusions		1 (4)	1 (4)
	Other indication for CVAD		2 (8)	0
CVAD type and subtype, *n* (%)	PICC line		2 (8)	4 (17)
	Subcutaneous port		23 (92)	20 (83)
		Port-a-Cath	7 (28)	12 (50)
		Vortex^®^	15 (60)	7 (29)
		Xcela Power^®^	1 (4)	1 (4)
CVAD site, *n* (%)	Jugular		23 (92)	20 (83)
	Brachial		2 (8)	4 (17)
CVAD duration, years median (range)			2.3 (0.2–8.9)	3.5 (0.3–8.5)
Use of VTE prophylaxis, *n* (%)	Prophylaxis at reduced dosing		-	10 (42)
	Prophylaxis at treatment dose		-	14 (58)
Type of VTE prophylaxis, *n* (%)	VKA		-	13 (54)
	LMWH		-	2 (8)
	ASA		-	4 (17)
	Rivaroxaban		-	1 (4)
	VKA and ASA		-	3 (13)
	Rivaroxaban and ASA		-	1 (4)
Additional VTE risk factors, *n* (%)	None		16 (64)	14 (58)
	Sepsis		1 (4)	1 (4)
	Pregnancy		3 (12)	1 (4)
	Prior VTE		3 (12)	9 (38)
	Medium-/high-risk surgery		1 (4)	1 (4)
	Line infection		2 (8)	5 (21)
Hydroxyurea as disease-modifying therapy, *n* (%)			17 (68)	12 (50)
Ferritin, mcg/L (median (range))			2608.0 (16–7908)	2114.5 (17–13065)
	Ferritin < 1000 mcg/L, *n* (%)		9 (36)	10 (42)
	Ferritin ≥ 1000 mcg/L, *n* (%)		16 (64)	14 (58)

Abbreviations: ASA = acetylsalicylic acid; BMI = body mass index; CVAD = central venous access device; LMWH = low molecular weight heparin; PICC = peripherally inserted central catheter; SD = standard deviation; VKA = vitamin K agonist; VTE = venous thromboembolism.

**Table 2:** Incidence rates of VTE according to the presence of thromboprophylaxis.

		Thromboprophylaxis			
		No (*n* = 25)		Yes (*n* = 24)	
Observation period, patient-years		62.2		86.2	
Events, type		Number of events	Incidence rate per 1000 implantation days (95% CI)	Number of events	Incidence rate per 1000 implantation days (95% CI)
	Any VTE *	10	0.44 (0.28–0.70)	4	0.13 (0.05–0.32)
	CRT	3	0.13 (0.05–0.38)	3	0.10 (0.03–0.28)
	PE	1	0.04 (0.01–0.30)	0	0
	Atrial thrombi	4	0.18 (0.07–0.43)	0	0
	Other ^&^	3	0.13 (0.05–0.38)	1	0.03 (0.005–0.22)

* One patient had 2 DVTs but is only counted once; ^&^ other sites included jugular vein, subclavian vein, and inferior vena cava. Abbreviations: CI = confidence interval; CRT = catheter-related thrombosis; DVT = deep venous thrombosis; PE = pulmonary embolism; VTE = venous thromboembolism.

**Table 3:** Univariate and multivariable Poisson log-linear regression. The presence of thromboprophylaxis, additional VTE risk factors, sex, and any variable with a *p*-value < 0.2 on univariate analysis were used to build a multivariable Poisson regression model of VTE incidence rates.

		Univariate (*n* = 49)			Multivariable (*n* = 49)		
Variable		VTE Rate Ratio	95% CI	Significance (*p*-Value)	VTE Rate Ratio	95% CI	Significance (*p*-Value)
Thromboprophylaxis							
	Present	1			1		
	Absent	4.0	(1.2–12.6)	0.02	14.9	(2.0–108.7)	0.01
Age categories							
	≥40 years	1			1		
	<30 years	0.3	(0.1–1.5)	0.15	13.4	(0.8–230.6)	0.07
	30 to <40 years	0.4	(0.1–1.8)	0.22	2.7	(0.2–38.1)	0.47
Sex							
	Male	1			1		
	Female	0.4	(0.1–1.2)	0.11	0.8	(0.1–4.9)	0.81
CVAD type							
	Subcutaneous port	1			-	-	-
	PICC line	1.8	(0.6–5.8)	0.32	-	-	-
CVAD location							
	Brachial	1			-	-	-
	Jugular	0.6	(0.2–1.8)	0.32	-	-	-
CVAD subtype							
	Conventional Port-a-Cath	1			1		
	PICC line	5.8	(1.3–25.9)	0.02	34.7	(3.5–343.6)	0.002
	Vortex and Xcela Power	58.2	(15.0–225.0)	<0.001	20.3	(3.8–107.9)	<0.001
BMI, kg/m^2^							
	BMI ≥ 25 kg/m^2^	1			1		
	BMI < 25 kg/m^2^	3.1	(1.0–9.8)	0.06	1.3	(0.1–18.6)	0.85
Ferritin (per additional mcg/L)							
	Ferritin ≥ 1000 mcg/L	1			-	-	-
	Ferritin < 1000 mcg/L	1.3	(0.4–4.8)	0.66	-	-	-
Additional VTE risk factors							
	Present	1			1		
	Absent	2.6	(0.9–7.6)	0.09	1.1	(0.2–4.9)	0.93
Hydroxyurea							
	Present	1			1		
	Absent	20.5	(6.4–65.3)	<0.001	47.1	(8.0–276.0)	<0.001

Only 4 patients with a non-SS/Sβ^0^ genotype were present in the study, precluding analysis. Abbreviations: BMI = body mass index; CI = confidence interval; CVAD = central venous access device; VTE = venous thromboembolism.

## Data Availability

The data presented in this study are available on request from the corresponding author. The data are not publicly available due to institutional restrictions.
